# Considerations for adapting digital competencies and training approaches to the public health workforce: an interpretive description of practitioners’ perspectives in Canada

**DOI:** 10.1186/s12889-024-21089-1

**Published:** 2025-01-10

**Authors:** Ihoghosa Iyamu, Swathi Ramachandran, Hsiu-Ju Chang, Andre Kushniruk, Francisco Ibáñez-Carrasco, Catherine Worthington, Hugh Davies, Geoffrey McKee, Adalsteinn Brown, Mark Gilbert

**Affiliations:** 1 School of Population and Public Health (SPPH), University of British Columbia (UBC), 2206 East Mall, Vancouver, BC V6T 1Z3 Canada; 2 BC Centre for Disease Control (BCCDC), Vancouver, BC Canada; 3 School of Health Information Science, University of Victoria, Victoria, BC Canada; 4 Dalla Lana School of Public Health, University of Toronto, Toronto, ON Canada; 5 School of Public Health and Social Policy, University of Victoria, Victoria, BC Canada

**Keywords:** Competency-based education, Professional competence, Digital public health, Health workforce, Health equity

## Abstract

**Background:**

Widespread digital transformation necessitates developing digital competencies for public health practice. Given work in 2024 to update Canada’s public health core competencies, there are opportunities to consider digital competencies. In our previous research, we identified digital competency and training recommendations within the literature. In this study, we explored public health practitioners' experiences and perspectives on adapting identified digital competencies and training recommendations for Canada.

**Methods:**

Between November and December 2023, we conducted an interpretive description using four focus groups with 19 public health practitioners working in regional and federal health authorities across Canada, with at least 3 years’ experience in current roles and experience using digital technologies in practice. We explored practitioners’ experiences using digital technologies and sought their opinions on how digital competency recommendations previously identified could be adapted to Canada’s context. To generate deep insights of practitioners’ subjective experiences and perspectives, we analyzed verbatim transcripts using Braun and Clarke’s reflexive thematic analysis.

**Results:**

We identified three main themes: a) public health systems must evolve to support new digital competencies; b) strengthen the basics before extending towards digital competencies; and c) focus on building general digital competencies with options for specialization where necessary. Findings emphasized matching workforce digital competencies to public health system capabilities and meaningfully integrating digital competencies within existing curricula. Such integration can consider how digital technologies change current public health practice to ensure practitioners are better able to address contemporary public health problems. Findings demonstrated roles for specialized digital programs as resources for learning within health systems and emphasized hands-on real-world training approaches.

**Conclusion:**

We need integrated, systems-focused approaches to digital competencies cutting across the current public health curriculum, while creating space for specialized digital public health competencies and roles. Further research is needed to understand requirements for enacting these recommendations in practice.

**Supplementary Information:**

The online version contains supplementary material available at 10.1186/s12889-024-21089-1.

## Introduction

Ongoing rapid digital transformations in public health and the broader society have increased demand for new digital competencies for the public health workforce [1, 2]. This is especially true since the COVID-19 pandemic when a plethora of digital tools were deployed to support public health functions [3, 4]. Public health organizations recognized the need for digital competencies to enable the workforce to leverage these tools for more proactive disease surveillance and response, enhance evidence-informed public health decision making (i.e., precision public health), more efficiently reach historically marginalized populations and increase effectiveness of public health services [3–6]. Digital competencies are also needed to help public health practitioners respond to contemporary public health challenges amplified by digital transformations in society, including misinformation and disinformation and the growing role of digital determinants of health and their intersections with social and commercial determinants [6–9]. For instance, a review of a data from 160 countries showed a link between social media disinformation and decreasing mean vaccination coverage and negative sentiment around vaccinations [10]. Studies have also demonstrated links between social media exposure and poor dietary habits, cardiovascular outcomes and cognition especially among children [11, 12].

Therefore, we conducted a three-phase study to identify digital competency and training recommendations for Canadian public health training programs. In phase one (rapid review) we found that, while there is acute awareness of the need for digital competencies in public health, few studies have made specific recommendations about competencies and training approaches necessary to support the digital transformation of public health [2, 13]. Where available, the evidence focused mainly on public health informatics. From a Canadian perspective, we found recommended digital competencies cut across all existing competency categories outlined by the Public Health Agency of Canada’s (PHAC) core competencies for public health in Canada framework [14]. We also identified competencies related to management and analysis of digital data streams using modern techniques, alongside new competencies regarding the management of informatics infrastructure necessary for digital data streams. An environmental scan of digital public health programs (phase-two) demonstrated curriculum and program development in response to widespread digital transformation is limited in Canada [2, 15].

Canada's public health system has unique characteristics that must be accounted for when conceiving digital competencies and training approaches to build these competencies [1]. Canada operates a federated and universal health system, with a mandate cutting across various jurisdictional systems organized differently across 10 provinces and 3 territories [1]. Publicly funded provincial and territorial health systems usually run through local and municipal health authorities, ministries of health and organizations which retain autonomy to adapt their operations and infrastructure to serve the needs of their local communities. While there is coordination from federal public health agencies like the Public Health Agency of Canada (PHAC), this structure introduces heterogeneity in terms of the infrastructure and workforce organization. Given Canada’s colonial history and commitment to truth and reconciliation, its public health systems focus on equity and Indigenous health [1, 16, 17]. Non-governmental, community based organizations and professional associations also contribute towards subject-specific public health goals that emphasize equity [1]. Canadian public health institutions offer undergraduate and graduate level training programs, alongside specialized programs like the Canadian Field Epidemiology Program and professional development and continuing education programs all guided by the PHAC Core Competencies for Public Health in Canada which was last published in 2008 [14]. However, in 2023, PHAC commissioned the National Collaborating Centres for Public Health to update the competencies [18]. Updates to the competencies should consider contextual factors necessary to garner public health practitioners’ support for these digital competencies including appropriate facilitatory conditions [19].

Given the emergent findings from previous phases, it is crucial to understand how competency and training recommendations identified from literature worldwide fit within the context of Canada’s public health system. Therefore, in this current study (phase-3 of the broader project), we aimed to explore how identified recommendations for competencies and training models for these competencies might be further adapted to Canada’s context to appropriately facilitate the digital transformation of public health. Here we conceptualized *digital competencies* as the essential knowledge, skills, and attitudes necessary to effectively use digital technologies for public health functions [14, 20, 21]. This definition extended beyond digital literacy (basic skills for using digital devices and applications effectively) to include technical skills for using digital technologies effectively in a work context and soft skills including understanding of ethics and equity implications of using these technologies in a public health context [22]. We asked: what adaptations may be required to apply the identified digital competency and training model recommendations to Canada’s public health training and practice context? and what competencies are required to ensure the public health workforce remains responsive to new digital technologies relevant to public health services in Canada?

## Methods

### Study design

This was an interpretive descriptive study that sought to ensure our research was aligned with experiences of public health practitioners affected by our recommendations (i.e., recommendations to public health schools and agencies involved in public health competency development) [23–26]. An interpretive description was appropriate given our aim to draw on existing knowledge and frameworks to inform better understanding of Canada’s public health context. We drew on Roger’s innovation diffusion theory and the Concerns-based adoption model to inform our inquiry including the development of discussion guides and our analyses [19, 27, 28]. We explored structural factors within the Canadian public health context that should be considered for successful implementation of training and explored participants’ concerns about the use of digital technologies within their practice. Reflecting on our motivations and experiences with this research, members of our team have experience implementing and evaluating digital public health interventions using equity-focused approaches. Team members are also educators with experience designing and evaluating public health training curricula at undergraduate and graduate levels. We sought to understand participants’ contexts assuming multiple realities and contexts [29].

### Study setting, sampling and participant recruitment

This study was conducted in collaboration with faculty at the: Dalla Lana School of Public Health, University of Toronto; School of Population and Public Health, University of British Columbia; and School of Public Health and Social Policy, University of Victoria. We recruited a stratified purposive sample of participants to represent perspectives of practitioners from western, central, eastern, and Atlantic Canadian regions and practitioners in federal and local public health agencies and health authorities. We sent recruitment emails using the research team’s networks to listservs of the BC Centre for Disease Control, Public Health Agency of Canada, The National Collaborating Centres, the Canadian Public Health Association, and other similar organizations across Canada. We included participants who were currently engaged in a federal, provincial/territorial, or municipal public health institution in Canada (including ministries of health and non-governmental organizations), in their current position for at least 3 years (to account for the time since the explosion of digital technologies to support the COVID-19 response) and familiar with using digital technologies in public health – either through practice in a decision-making capacity or as a frontline public health practitioner required to work with digital technologies. We excluded participants who were interested but had no experience with digital technologies or served in an administrative capacity in health systems, with roles restricted to a solely clinical perspective (i.e., focused on “digital health” which involved digital technologies in clinical settings like personalized health records and health apps) [30].

### Data collection

For consenting participants, we sent collected demographic and professional information before focus groups. Four focus groups (FGs) were conducted online using Microsoft Teams® between November 7th and December 7th, 2023, each lasting an average of 100 min (range 84–111 min). Focus groups were created based on participants’ geographical location and organizational jurisdictions (Table 1). Participants took these calls alone in private office spaces or in their homes. We followed a discussion guide (Appendix 1) which was broadly divided into two sections. While none of the interviewers had prior relationships with FG participants, we began FGs by having broad conversations about the goals of the study, the study team and then engaged in a discussion of participants’ broad perceptions and experiences of digital competencies in their current practice. Then we reviewed a list of sample competency statements generated from a rapid review of digital competencies for public health (Phase 1 of the broader study—Appendix 2) [2]. During this review, we invited comments and discussions from participants about how the proposed competency statements applied or did not apply given their experience with public health in Canada. FG guides were not piloted but were repeatedly reviewed by members of the research team who have extensive experience of the Canadian public health context. All FGs were conducted by II (a physician and public health researcher with 5 years of experience and training in qualitative methods including interviewing and FG facilitation). SR and CW made detailed field notes and co-facilitated FGs as appropriate. We audio recorded FGs using the record function on Microsoft Teams® and created verbatim transcripts using the live translate function. To ensure accuracy, one of the researchers (SR) reviewed live transcripts with the audio recordings, while a second researcher (II) checked the corrected transcripts. We also collected detailed field notes describing participants’ non-verbal and verbal cues within the group and our general impression of the group dynamics. All study materials were stored on encrypted servers.

**Table 1:** Focus groups and participant composition

**Focus Group**	**Composition**	Number of participants
**Group 1** Regional/Provincial and local public health practitioners^a^	Public health practitioners from municipal and regional health authorities in Eastern and Central Canada.^b^	5
**Group 2** Regional/Provincial and local public health practitioners^a^	Public health practitioners from municipal and regional health authorities in Western Canada.^c^	4
**Group 3** Regional/Provincial and local public health practitioners^a^	Public health practitioners from municipal and regional health authorities in Western Canada.^c^	4
**Group 4** Federal and other interjurisdictional public health practitioners	Public health practitioners from Indigenous Services Canada and other federal organizations serving on equity-seeking populations	6

^a^Geographic distinctions were created to explore differences in thinking about digital competencies for public health. Our previous studies suggest more potentially relevant programs and courses have been developed in Central and Atlantic Canada compared with Western Canada

^b^Ontario, Quebec and Atlantic Canada

^c^BC and Alberta (combined for convenience given small sample)

### Ethics

We obtained ethics approval from the University of British Columbia’s Behavioral Research Ethics Board (ethics #H22-03153). We obtained written voluntary informed consent from each participant at least 24 hrs prior to FGs using Qualtrics and assigned each participant an identification number (ID) which was included in all FG transcripts and field notes. All study materials were stripped of personal identifiers prior to analyses. Each participant was offered a $50 CAD honorarium and study protocols adhered to the principles and requirements laid out in the Declaration of Helsinki [31].

### Data analysis

FG transcripts, demographic data, and field notes were imported into QSR NVivo version 14 for data management and analysis. We conducted reflexive thematic analyses following Braun and Clarke’s recommendations, as this approach effectively explores complex experiences and perspectives, providing the nuanced insights necessary to inform potential updates to digital competencies [32–34]. First, SR and II comprehensively read the transcripts and noted general ideas from the transcripts before conducting line by line inductive coding which was led by SR. Both analysts intermittently met to discuss the codes. Thereafter, II reviewed initial codes and categorized the codes, applying concepts from selected theories as applicable. This followed an inductive-deductive process culminating in a codebook that both analysts reviewed for clarity. Based on the initial categories, we identified relationships and shared meanings to create preliminary themes which were presented to the research team, reviewed and edited to create a final list of themes. Treating each FG as the unit of analyses, we explored differences in group perspectives on digital competencies and adaptations required in a Canadian public health context. Throughout the analyses, II and SR made reflexive memos about our perspectives of the data. Feedback from the research team informed our process for clarifying interpretations of the themes before finalizing and drafting the report. Research reporting adhered to the consolidated criteria for reporting qualitative studies (COREQ) (Appendix 3) [35].

## Results

We conducted four focus group discussions with 19 public health practitioners representing regional, provincial, and federal public health institutions across Canada (Table 2), mostly from British Columbia and Ontario. Among participants, 74% self-identified as women, 37% were 31–40 years old, 47% worked in frontline public health roles including public health nurses and public health physicians and 37% had between 3–5 years of experience in their current roles (Table 2).

**Table 2:** Focus group participants' characteristics

**Characteristic**	**N (%)** 19 (100.0%)
**Age (years)**	
26–30	3 (15.8)
31–40	7 (36.8)
41–50	3 (15.8)
50 +	5 (26.3)
Not specified	1 (5.3)
**Gender Identity**	
Man	3 (15.8)
Non-Binary	1 (5.3)
Not specified	1 (5.3)
Woman	14 (73.7)
**Role in organization**	
Public health nurse/physician/inspector	9 (47.4)
Health promotion specialist	2 (10.5)
Manager	3 (15.8)
Others	5 (26.3)
**Jurisdiction/type of public health organization**	
Federal public health agency^a^	6 (31.6)
British Columbia regional health authority	7 (36.8)
Ontario regional health authority	4 (21.1)
Other regional health authorities	2 (10.5)
**Experience in current role (years)**	
3–5	7 (36.8)
6–10	5 (26.3)
11–15	3 (15.8)
16–20	1 (5.3)
20 +	3 (15.8)

^a^Includes agencies serving First Nations, Inuit, and Metis communities

**Fig. 1:**
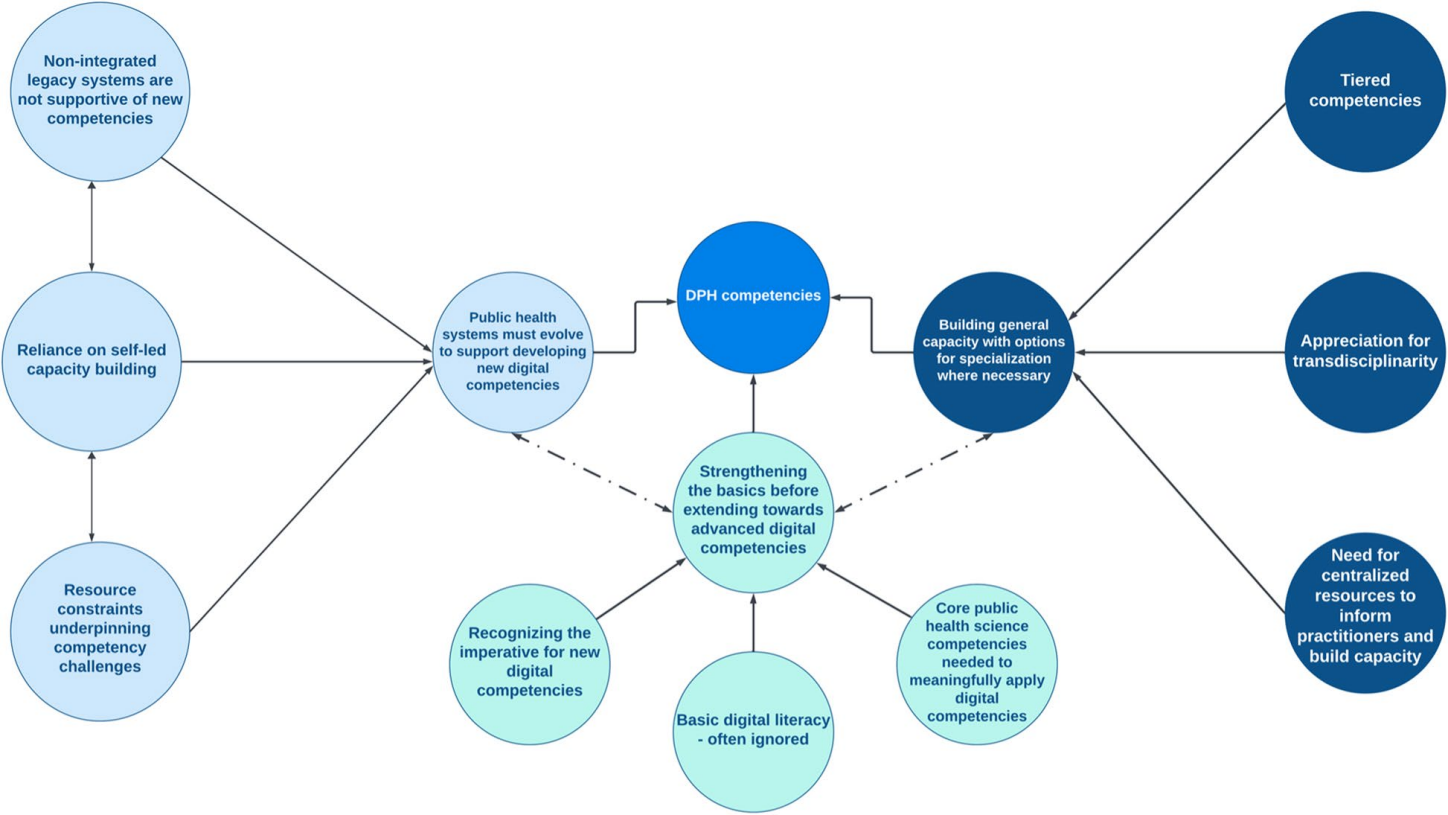
Map of themes identified in focus groups with public health practitioners discussing digital competencies for public health; DPH: Digital public health. (Dashed lines represent relationships between main themes identified)

We identified three main themes in the focus group discussions (Fig. 1). Below, we describe the themes in detail, including exemplary quotes where appropriate:

### Public health systems must evolve to support new digital competencies

Public health practitioners realized and suggested that, for digital competencies to be beneficial, digital public health systems must effectively facilitate their enactment. Practitioners described their work situations where a fragmented and issue-based approach to implementing digital systems has resulted in non-integrated (non-interoperable) legacy systems that obstruct the broad vision for digital transformation of public health systems. These views were shared in all the focus groups representing organizations in Western, Eastern, and Central Canada. Practitioners provided multiple examples of legacy systems co-existing alongside modern alternatives where health workers are required to manually enter data from one system into others, or persistently using paper-based records despite existence of digital alternatives. For example, a 31 to 40-year-old health promotion specialist from Ontario described the confusing digital systems required to track respiratory infections:

*“During COVID, it was all [software1 name] in Ontario all the time…But now that we have other communicable diseases to work with as well, integrating [software1 name] with our [software2 name], which is just another provincial database where we enter communicable diseases is very difficult because COVID goes into one database, everything else goes into [software2 name].. In addition to that, inspectors use their own database called [software3 name], which is probably the most widely used in Ontario with the health units… and it doesn't integrate with anything…. So how do we get everyone on board with everything? So now we have three separate databases that we're trying entry into.” – FG 1.*

While these parallel systems require multiple trainings and retraining, most practitioners described self-led capacity building as the most common approach to gaining digital competencies for public health. Self-led capacity building was often described as driven by personal interest among early adopters and digitally native (i.e., gaining technological savviness because they grew up with technology around them) practitioners. Being digitally native was described more regarding younger practitioners, acknowledging the workforce’s diversity and the potential for digital technology to be overwhelming for others who may be unwilling to build capacity. A 50 + year old public health nurse in Ontario stated:

*“Very few of our nurses have [the digital competency], even because of our age, you know. I'm one of the few that is willing to go back to school and get the database training and get the word processing training and umm, they [other older public health practitioners] don't even know how to do word processing.” – FG1.*

Practitioners recognize resource constraints are partly responsible for challenges described and emphasize difficulties maintaining a stable workforce as a contributor to competency challenges. They suggested that frequent staff turnover has resulted in a workforce that is unable to work with the evolving data systems despite ongoing training and mentorship. Practitioners noted that suboptimal resourcing influences the non-integrated systems as digital systems are built with suboptimal funding while also navigating the digital divide (i.e., inequitable access to and use of digital technologies) and other structural barriers (e.g., privacy regulations and resistance to use) that limit digital access between teams and among program beneficiaries. Generally, most participants recognized the imperative for building new digital competencies to keep up with changing public expectations of public health services and digital transformation of society. This transformation was said to influence how different populations are better reached through different digital platforms and comparative efficiencies in times savings for public health workers and the public with client-led service access through digital platforms.

### Strengthen the basics before extending towards advanced digital competencies

Yet, practitioners suggested we must prioritize basic competencies first, before proceeding to specialized digital competencies. While specialized digital competencies were used to describe advanced competencies like advanced data science and public health informatics that would not be required of practitioners in general practice, the basics were described in two perspectives. The first involved strengthening core public health competencies to ensure meaningful application of digital competencies. For example, practitioners described needing a combination of basic understanding of biostatistics and public health sciences, deep institutional knowledge of digital data generation processes, and communication skills to effectively interpret and translate digital data streams and analyses into insights that benefit public health. A public health inspector from Alberta said:

*“You have to as the so called you know information person have to be able to have a deep understanding of how the data is formed and your ability to communicate it… You have to be able to do both of those things and a lot of people aren't.” – FG3.*

These discussions emphasized multiple combinations of competencies that practitioners must harness to make sense of contemporary public health practice. Similar examples were provided for program planning, implementation, and evaluation, as well as health promotion and equity. For health promotion, practitioners described core public health competencies related to partnership and communication to engage diverse communities using specially created content via social media. However, most groups recognized the importance of competencies related to the ethical management of digital data, especially regarding understanding privacy risks and mitigation measures to appropriately use the data for decision making while prioritizing equity. Practitioners described variations in competencies between the public health workforce in communities and those working in regional, provincial, and federal organizations. For example, a 40–50-year-old nurse manager working at a federal organization suggests:

*“The challenge if we are looking at the evolution of technology and with the evolution of technology, we have an evolution of hackers. To be honest, they understand those systems as well. So, I think there should be a level of protection around these types of systems and access of individuals who are using these systems… I'm not entirely sure about a provincial level, but you know, and then when you look at in community, there really isn't a whole lot of understanding around the data, systems, governance and management”* – FG4.

Second, practitioners described the need for basic digital literacy skills like being able to use enterprise tools including Microsoft Office suite and other similar tools required for the public health workforce’s daily activities. They noted that most of the health workforce is unable to use many of the available features and only manage with features required to successfully do their work at a minimum. Practitioners noted the diversity of the public health workforce with generational differences in experience and dexterity with using digital tools required for their public health functions. This was described as an often-ignored issue with the growing population of older health workers like public health nurses who have greater difficulty using enterprise tools and devices compared with younger counterparts. Practitioners did not necessarily describe this as negatively influencing public health outcomes as older health workers were conversely described as having greater understanding of core public health competencies compared with younger workers. A nurse advisor at a 50 + year-old federal organization said:

“*As a result of the pandemic, the recruitment and retention are just horrible and I know they've got a strategy in place or supposedly, but I mean even for the complement of nurses that I teach, they're borrowing and stealing to try and get someone in place… I've trained nurses that are in their 70 s on a computer system and it's, you know, it has its own set of challenges, right? As opposed to the younger generation that are coming out that are very tech savvy and can navigate systems pretty quick. So that's definitely a huge thing.” –* FG4.

### Focus on building general digital competencies with options for specialization where necessary

Practitioners recognized the importance of team-based approaches to public health, knowing that their understanding of competencies required of their role will be varied and limited in different circumstances depending on their roles within the team. They also described the importance of having a generalized understanding of how digital technologies work in relation to public health functions, but not necessarily being able to apply such skills in specialized roles. Emphasis should be on the core of public health functions while considering how digital technologies might affect or help facilitate the public health services. For example, a 30 to 40-year-old epidemiology and informatics manager in Ontario said:

*“It's sort of like, you know what is core and what is not… Just so you know, we're gonna do a community flu clinic. Like how should we promote it? What should be digital? But we have patient self-scheduling. How are we gonna do record keeping? Should digital be considered? What's the core of that is a flu clinic in my opinion. Like the digital is a tool that helps enable us to do the core of it [which] is the public health service…”* – FG1.

Practitioners’ suggestions highlighted the need for tiered digital competencies, acknowledging that public health is a transdisciplinary practice with room for specialized digital competencies where necessary (Appendix 2). However, they suggest a generalized understanding can help with communication between teams with varied perspectives on public health operations.

Practitioners further described the importance of centralized resources and resource persons who deeply understand digital technologies within organizations, are engaged with the trends in development of digital technologies in public health and can disseminate these learnings within public health organizations and across communities of practice. From an individual standpoint, practitioners highlighted important skills and attitudes such as curiosity, willingness to ask questions and continuous learning as important skills and attributes needed from public health practitioners who must keep pace with the development of digital technologies for public health. However, they highlighted the need for systems to support these attributes.

Finally, practitioners suggested that the most effective training approaches must include applied learning to allow trainees to engage with real-world challenges navigating digital technologies in public health practice. These could include using case studies and applied learning through internships and practicums rather than perfectly prepared classroom settings and cases that do not help trainees understand the complexities of applying digital competencies in real-world practice. One 30 to 40-year-old epidemiologist from British Columbia described this in detail:

*“Personally, [in] my experience, I felt like everything I learned, though it makes sense, it's always in the ideal situation. Ideal scenarios, perfectly clean data for me to analyze. Everything is very perfect already, so it felt like it didn't really prepare me when I came into a system where there's already a lot of potential issues or things that need to be resolved. So, a program or something that deals… [with] case study related [training], something that makes you think, or you know develop these kinds of problem-solving skills and not necessarily thinking everything given to you... You know already clean data, and everything is just ready for full functioning.”* – FG2.

## Discussion

We explored how recommendations for digital competencies and training models might be adapted to Canada’s public health systems and competencies required to ensure a workforce that responsively adapts new digital technologies. We found that practitioners recognize the importance of digital competencies and training with the discourse centered on three themes. First, public health systems must evolve to support new digital competencies, emphasizing the need for parallel advocacy efforts to strengthen the digital capabilities of public health systems while building digital competencies among practitioners. Second, we must focus on strengthening the basics before extending towards more advanced digital competencies. Here, practitioners emphasized fundamental public health competencies and digital literacy including using basic tools for word processing and other similar processes as priority areas. Third, we must focus on building general digital competencies (beyond digital literacy) with options for specialization where necessary. This theme demonstrated the need for high-level understanding of various digital aspects of public health domains, with opportunities to further develop digital competencies in specialized roles as needed.

While increased attention to digital competencies resulted from the COVID-19 pandemic, studies systematically exploring these competencies and training approaches needed for contemporary public health practice are sparse [1, 2]. This is more apparent for public health workers who carry out a broad range of functions to ensure health for all [13, 36]. Recent reviews of public health training curricula show this is a gap requiring urgent attention [37]. Where available, evidence corroborates our findings about the need to strengthen basic digital literacy to enable public health practitioners’ use of modern digital tools for public health functions [36]. Our findings demonstrate the relevance of basic digital literacy given the diversity of the public health workforce, with younger practitioners considered as digital natives, while many older practitioners inequitably struggle with digital systems [36]. This is often overlooked in current discourse.

This study adds considerations regarding digital competencies for the Canadian public health workforce. First, our study emphasizes the importance of maintaining a systems approach to digital competencies. The Australasian digital health capability framework explores somewhat similar standards assuming professional upskilling must occur alongside efforts to build organizational digital capabilities in a more cohesive manner [38]. We extend this thinking to include concurrent efforts to build digitally mature health systems alongside workforce digital capabilities [39]. Our study demonstrates gaps in current staccato approaches to building competencies through self-led capacity building which result in significant waste as these self-built competencies are limited by systems not built to leverage new digital competencies [40]. Our study also adds considerations for more meaningful integration of digital competencies within curricula, emphasizing the need to explore digital technologies from the perspective of public health, identifying opportunities to leverage digital tools for public health functions, while considering potential risks that digital tools across society might pose to the effective operationalization of public health functions [8, 30].

Findings demonstrate the importance of specialized digital competencies for specific public health practitioners who lead digital transformations, maintain currency about digital tools and innovations in public health, and inform interdisciplinary and transdisciplinary teams about potential opportunities, alongside a more general understanding of the role of digital technologies among practitioners in a more general setting. Finally, our findings corroborate other studies that suggest applied learning through practicum and case studies can more appropriately build digital competencies in contemporary practice [41].

### Implications for research, funding, policy and practice

There are significant implications from this study given current efforts to update the 2008 public health core competency framework in Canada [42]. We discuss implications from the health systems and education perspectives.

*Implications for health systems:* our findings emphasize digital competencies as part of the broader discourse around digital maturity of public health systems and encourage careful matching of systems capabilities with upskilling efforts within the public health workforce [39, 43]. This suggests public health practitioners and decision-makers must engage longstanding challenges with current approaches to implementing digital systems for public health that are often fragmented and issues-based [30, 40]. The Australasian Digital Health Capability framework is an example of first steps in this direction, exploring a systems approach to building skills to function in an increasingly digital environment [38]. We have previously described the lack of a clear digital public health strategy as a gap in Canada [30]. Such a strategy might benefit from a systems approach to building competencies alongside digital capabilities of the health systems.

Moreover, public health organizations must partner with schools to strengthen offerings for basic digital literacy training (i.e., skills to use enterprise software and devices), acknowledging the growing diversity of the public health workforce especially in terms of age and other social factors and their varying levels of digital technology adoption [36]. Given target audiences for training, these offerings could be made available as micro-credentialing or other continuing education options. Such digital literacy options can be entry points into considering more integrated public health digital competencies as previously described and can ensure the public health workforce properly utilizes enterprise tools to improve operational efficiencies. This will be critical if the public health workforce aims to promote equity and reach underserved populations most affected by current systems-inefficiencies [44, 45].

*Implications for schools of public health*: our findings highlight the need for two main approaches. First, schools can adopt a public health-first approach that integrates considerations for digital technologies across the existing curricula. We must consider how digital technologies can be leveraged to optimize delivery of specific public health functions and ensure competencies with these tools, while increasing awareness and competencies to manage threats posed by widespread use of digital technologies. For example, regarding health promotion, public health curricula must integrate digital concepts into health promotion courses to build trainees’ competencies with understanding roles that social media can play in targeting specific audiences, evaluating differences in public health messaging strategies across various media formats, and being aware and equipped with tools to actively monitor and address the risk of online mis- and disinformation within communities they serve [46, 47]. Such curricular approaches will require exposure to various disciplines, while deepening public health science knowledge. Similar arguments have been made for “T-shaped” and other similarly conceived professional trainings promoting deep knowledge in specific practice areas while strengthening capacity to understand and engage a broad range of disciplines in team-based transdisciplinary practice [48, 49]. A public health first approach will promote compatibility of digital content with current public health curricula, ensuring better uptake as described in Roger’s innovation diffusion theory [27, 28]. However, with already packed public health curricula, the volume of digital content integrated may be limited. Organizational policies including limited approvals for usable social media platforms, and current practices supporting social media in traditional “one-way” communication must be addressed to align digital capabilities of systems with workforce digital competencies [50].

Second, public health curricula need to create tracks for specialized digital competencies as applied in public health. These specialized roles can include advanced data analytics using modern approaches like artificial intelligence and machine learning approaches grounded in biostatistics principles, public health informatics, infodemic management, and human-centered design amongst others [2]. There are already examples of such specialized tracks in schools of public health globally, but very few within the Canadian context. Given findings about the need for these specialized roles serving as centralized resources with digital expertise within organizations, such programs need to integrate competencies for lifelong learning and curiosity, ensuring continuous awareness new digital technologies applied in public health [38].

Considering implications for generalized and specialized digital competencies, tiering competency statements appears to be a logical suggestion from practitioners. Practitioners suggest having a general understanding of most digital competencies with increasing levels of competence based on levels of expertise and engagement with the subject areas. Similar approaches have been implemented in competency frameworks by the Council on Linkages Between Academia and Public Health Practice and the Association of Schools of Public Health in the European Region [51, 52]. We have made similar adaptations to previously identified competency statements (Appendix 2), accounting for practitioners’ expertise levels and depth of engagement with digital competencies [2].

There is a crucial need for real-world hands-on experience with digital tools applied in public health functions [41]. Trainees need to be exposed to the messiness of real-world settings in terms of wrangling data from multiple sources, and engaging project partners to design and implement digital public health interventions. However, it will be necessary to understand the pragmatic implications for doing this including time for coursework and the necessary partnerships between schools of public health and public health institutions with opportunities for hands-on training [42]. Despite a range of concerns raised by public health practitioners, many of our study participants were in the management stage of the concerns-based adoption model where expressed concerns regarded the logistics of implementing digital competencies within currently limited public health systems. In addition to a systems-approach to digital competencies, training recommendations to address this stage of the concern include integrating such digital competencies into existing public health training and establishing mentorship especially through transdisciplinary support networks and communities of practice [27, 53]. Here, we define transdisciplinary networks to include collaborations beyond traditional partnerships in public health, blending approaches from physical sciences, computer sciences, behavioral sciences, communications and other similar disciplines to create unique solutions to public health problems [54]. Examples of training programs adopting these transdisciplinary cohort-based supporting systems includes McGill’s dual degree in public health data science that builds student triads that deepen experiences of public health systems and allows students to develop skills to engage in a transdisciplinary workforce.

Additional research is needed to understand faculty perspectives about needed digital competencies and current barriers to implementing suggestions and recommendations outlined in this study. We acknowledge that integrating digital competencies in the curriculum requires significant upskilling among public health faculty and formation of transdisciplinary partnerships. Research is required to understand faculty concerns with designing and delivering such curricula, evaluate the level of training required for public health faculty and to determine the configuration of partnerships needed to optimize learning environments. While we have provided initial recommendations, additional competencies may need to be considered in contemporary public health curricula to address emergent technologies, such as generative artificial intelligence and other similar modern tools.

### Strengths and limitations

This study explored pan-Canadian perspectives through focus groups with practitioners across Western, Eastern, and Central Canada, working at regional, provincial and federal health authorities and agencies. This broad perspective ensured our study findings account for the differing attributes of public health systems across Canada and provided practical insight about potential changes necessary for public health training to keep up with contemporary practice. Our team includes education leads at schools of public health in Canada to help ensure our interpretations were grounded in their reality. However, we were limited by the number of focus groups we could conduct per region and organizational characteristics and by the distribution of participant roles within focus groups. While our sampling strategy allowed us to reasonably explore the breadth of codes (issues) related to our topic of interest, we were unable to sufficiently gain depth and meaning saturation that routinely requires two or more groups per strata [55]. Further, we were limited by the sample size and could not sufficiently explore regional differences in perspectives. This is important given regional differences in the organization of public health services in Canada. While having 74% of the sample as women is representative of the gender distribution of the Canadian public health workforce, we are somewhat limited in our understanding of the experiences of men and gender diverse populations within the workforce [56]. However, we remain confident that current recommendations will be broadly relevant in most regions given commonalities between various Canadian public health organizations, training programs, and schools of public health.

## Conclusion

Public health practitioners recognize the importance of digital competencies in contemporary practice and the need to train for these competencies. However, to be successfully implemented in public health curricula and to have meaningful public health impact, there must be concurrent efforts to develop capacity of public health systems to accommodate new digital competences. Efforts to adapt digital competencies for Canada’s public health systems must acknowledge the age diversity of the workforce, focusing on building digital literacy and integrating digital competencies in current formal degree awarding program curricula using a public health-first approach that considers how digital technologies can be leveraged or can affect current public health practice. Such integration efforts should accompany opportunities for continuing education. Where applicable, there is room for specialized or expert digital competencies with considerations for competencies that foster life-long learning. These competencies will require real-world hands-on training and transdisciplinary partnerships across schools of public health in Canada. Further research is required to understand requirements to enact current recommendations especially from faculty perspectives.

## Supplementary Information

Supplementary Material 1.

## Data Availability

The datasets used and/or analyzed during the current study are available from the corresponding author on reasonable request.
