# Supplementary material for: Considerations for adapting digital competencies and training approaches to the public health workforce: an interpretive description of practitioners’ perspectives in Canada
Source: BMC Public Health. 2025 Jan 10;25:122. doi: 10.1186/s12889-024-21089-1 (PMC11720584; doi:10.1186/s12889-024-21089-1)
Supplement: Supplementary file 1 — Supplementary Material 1. [file 12889_2024_21089_MOESM1_ESM.docx]

# **Appendices**

## *Appendix 1 – Topic guide*

1. Welcome – participant introductions and ground rules.
   1. CONFIRM THAT EVERYONE HAS SIGNED INFORMED CONSENT.
   2. ENCOURAGE EVERYONE TO SPEAK FREELY as this is not an evaluation of themselves or their programs but to gain insight into ways to adapt identified digital competency recommendations and training models based on your experiences.
   3. ENCOURAGE PARTICIPANTS TO WAIT FOR THEIR TURN BEFORE SPEAKING – can use the “raising hand” function on Microsoft Teams.
   4. ENCOURAGE PARTICIPANTS TO ASK EACH OTHER QUESTIONS AND COMMENT ON OTHERS’ DISCUSSIONS IN A RESPECTFUL MANNER.
   5. REVIEW THE RECOMMENDATIONS AT A HIGH LEVEL – focusing on the first one or two recommendations per competency category and the list for the new suggested competency category. ALSO REVIEW TRAINING RECOMMENDATIONS.
2. How relevant are these digital competency recommendations [LIST SHARED TO PARTICIPANTS] to the existing competency framework?
   1. Given your experiences with digital technologies in public health, which of the reviewed competencies resonate with you and why [ASK AS A ROUND-ROBIN AND ICE BREAKER TO GET EVERYONE INVOLVED IN SPEAKING – ENCOURAGE PARTICIPANTS TO GIVE EXAMPLES OF THEIR EXPERIENCES WITH COMPETENCIES THEY IDENTIFIED AS IMPORTANT]?
   2. What digital competency recommendations would be necessary for every professional practicing public health in Canada? Please explain why.
   3. Which of the digital competency recommendations would be more suitable for specialized public health roles in Canada?
   4. What factors within the Canadian public health training and practice context have influenced the need for identified digital competency recommendations [GIVE EXAMPLES BASED ON PREVIOUS CONVERSATIONS IN 2A.]?
   5. Are there any digital competencies listed that might not be useful within a Canadian public health context? Please explain why.
   6. What concerns do you have regarding the digital competency recommendations presented to you today, especially given your perspective of public health in Canada?
3. How might we appropriately train the public health workforce to meet the identified digital competency recommendations?
   1. What experiences have you had with designing and implementing training programs to build public health workers’ capacity to use digital technologies?
   2. Given the digital competency recommendations that resonate the most with this group, what training models will be most suitable to build capacity for these competencies?
   3. Are there any barriers to implementing suggested training models? Please explain the barriers.
   4. What types of disciplines would be required to facilitate suggested training models?
   5. What benefits and challenges are there with integrating partnerships with suggested disciplines to implement the training models?
4. Given that new digital technologies are constantly being developed and deployed in public health, what competencies would be required to ensure public health practice remains adaptable to the integration of new digital technologies within the practice?
5. Are there any other comments or concerns you would like to share with us?

##

## *Appendix 2 – Original list of competency and training recommendations reviewed and adapted by focus group participants*

| **Competency category** | **ID** | **Original Competency statements^$^** | **Tier 1 - Expert (Role requires working with competencies in designing, implementing and evaluating digital systems and supervising teams to utilize such systems)** | **Tier 2 - Proficient (Role requires understanding of digital systems and their connections with core public health functions. May not directly design, implement and evaluate digital systems but leads cross-functional and interdisciplinary teams that have expertise with the systems)** | **Tier 3 - Competent (Role requires engaging directly with core public health competencies but must understand how digital systems provide opportunities or may influence public health functions and outcomes)** |  |
| --- | --- | --- | --- | --- | --- | --- |
| **Public Health Sciences** | **A.1** | •      Develop and ethically apply research methods including data science, statistical genetics & omics technologies (i.e., exposomes), computational biology, epidemic and infectious disease modeling to public health problem. | •      Develop and ethically apply research methods including data science, statistical genetics & omics technologies (i.e., exposomes), computational biology, epidemic and infectious disease modeling to public health problems. | •      Understands processes and personnel required to develop and ethically apply research methods including data science, statistical genetics & omics technologies (i.e., exposomes), computational biology, epidemic and infectious disease modeling to public health problem. | •      Understands ethical principles guiding research methods including data science, statistical genetics & omics technologies (i.e., exposomes), computational biology, epidemic and infectious disease modeling to public health problem. |  |
|  | **A.2** | •      Apply systems thinking to public health issues. | •      Apply systems thinking to public health issues. | •      Apply systems thinking to public health issues. | •      Apply systems thinking to public health issues. |  |
|  | **A.3** | •      Use different types of data to answer public health questions. | •      Design, implement, and evaluate the use of diverse data types to answer complex public health questions. | •     Lead teams to understand and utilize various data types to address and answer complex public health questions. | •      Understands how diverse data types can be used to answer complex public health questions. |  |
|  | **A.4** | •      Aid public health organizations in thinking through, designing and testing new (digital) systems. | •      Designs, tests and implements new (digital) systems in public health organizations. | •      Leads public health organizations in thinking through, designing, testing and implementing new (digital) systems. | •      Aid public health organizations in thinking through, designing and testing new (digital) systems. |  |
|  | **A.5** | •      Conduct education and training in public health informatics. | •      Conduct education and training in public health informatics. | •      Understands education and training needs for public health informatics and leads training efforts. | •      Participates in personal education and training needs for public health informatics. |  |
| **Assessment and analysis** | **B.1** | •      Use, protect and interpret complex, linked large data sets from multiple sources and across levels of organization from administrative, clinical, biologic, environmental, population to social/societal levels, within and outside the health systems. * | •      Use, protect and link large data sets from multiple sources (e.g., administrative, clinical, biologic, environmental, population and social/societal levels) within and outside the health systems. | •      Understands processes for linking, using and protecting complex large data sets from multiple sources (e.g., administrative, clinical, biologic, environmental, population and social/societal levels) within and outside the health systems. | •      Is aware about data linkage processes and protocols to protect large data sets from multiple sources (e.g., administrative, clinical, biologic, environmental, population and social/societal levels) within and outside the health systems. |  |
|  | **B.2** | •      Design, analyze and report health science data using a blend of traditional and modern analytic and computational techniques (e.g., biostatistics, informatics, computer-based programming, and software, as appropriate - Tableau, python, MS-SQL​, R). | •      Design, analyze and report health data using a blend of traditional and modern analytic and computational techniques (e.g., biostatistics, informatics, computer-based programming, artificial intelligence, and software, as appropriate - Tableau, python, MS-SQL​, R). | •      Understands the design, analyses and reporting of health data using a blend of traditional and modern analytic and computational techniques (e.g., biostatistics, informatics, computer-based programming, artificial intelligence, and software, as appropriate - Tableau, python, MS-SQL​, R). | •      Analyze and report health data using a blend of traditional and modern analytic and computational techniques (e.g., biostatistics, informatics, computer-based programming, artificial intelligence, and software, as appropriate - Tableau, python, MS-SQL​, R). |  |
|  | **B.3** | •      Identify needs, challenges, principles, and key details for data sharing, challenges of large-scale data management. | •      Identify and understand data sharing needs and principles and address challenges to large-scale data use in public health systems. | •      Understands data sharing needs and principles and coordinates transdisciplinary teams to address challenges to large-scale data sharing and use in public health systems. | •     Understands data sharing needs, principles and limitations in public health systems. |  |
|  | **B.4** | •      Know and understand methodological and statistical problems inherent in the analysis of secondary and big data. | •      Understand and address methodological and statistical problems inherent in the analysis of secondary and big data. | •      Understand methodological and statistical problems inherent in the analysis of secondary and big data. | •      Is aware of methodological and statistical problems inherent in the analysis of secondary and big data. |  |
|  | **B.5** | •      Use and interpret findings from data exploration tools and other analytics. | •      Generate and interpret findings from data exploration tools and other analytics. | •      Interpret findings from data exploration tools and other analytics. | •      Interpret findings from data exploration tools and other analytics. |  |
|  | **B.6** | •      Use information technologies and communication tools necessary to support epidemiologic investigations and surveillance - e.g., smartphone survey, GPS tracking. | •      Use information technologies and communication tools necessary to support epidemiologic investigations and surveillance - e.g., smartphone survey, GPS tracking. | •      Understand the use of information technologies and communication tools necessary to support epidemiologic investigations and surveillance - e.g., smartphone survey, GPS tracking. | •      Recognizes opportunities to use information technologies and communication tools necessary to support epidemiologic investigations and surveillance - e.g., smartphone survey, GPS tracking. |  |
|  | **B.7** | •      Understand how "omic" tools can be integrated into an ecological model of health including social and environmental determinants. | •      Understands and integrates "omic" tools into an ecological model of health including social and environmental determinants. | •      Understands how "omic" tools can be integrated into an ecological model of health including social and environmental determinants. | •      Is aware about the use of "omic" tools and their integration into an ecological model of health. |  |
|  | **B.8** | •      Identify the role of emerging "omics" technologies applicable to epidemiologic research and incorporate them into epidemiologic research. | •      Identifies the role of emerging "omics" technologies applicable to epidemiologic research and incorporates them into epidemiologic research. | •      Understands the role of emerging "omics" technologies applicable to epidemiologic research. | •     Is aware about the role of emerging "omics" technologies applicable to epidemiologic research. |  |
|  | **B.9** | •      Understand general methods for linking data resources for creating information. | •      Understands general methods for linking data resources for creating information. | •      Understands general methods for linking data resources for creating information. | •      Is aware about general methods for linking data resources for creating information. |  |
|  | **B.10** | •      Design, implement and evaluate population-based projects, programs or interventions that use social media as a communication platform and a tool for public health education and promotion. | •      Design, implement and evaluate population-based projects, programs or interventions that use social media as a communication platform and a tool for public health education and promotion. | •      Understands processes necessary for the design, implementation and evaluation population-based projects, programs or interventions that use social media as a communication platform and a tool for public health education and promotion. | •      Design, implement and evaluate population-based projects, programs or interventions that use social media as a communication platform and a tool for public health education and promotion. |  |
| **Policy and program planning, implementation, and evaluation** | **C.1** | •      Develop tools that protect the privacy of individuals and communities involved in health programs, policies, and research. | •      Develop and apply tools that protect the privacy of individuals and communities involved in health programs, policies, and research. | •      Leads interdisciplinary teams to develop tools that protect the privacy of individuals and communities involved in health programs, policies, and research. | •      Applies tools that protect the privacy of individuals and communities involved in health programs, policies, and research. |  |
|  | **C.2** | •      Support use of informatics to promote disease prevention at the clinical health, environmental and personal health interface. | •      Support use of informatics to promote disease prevention at the clinical health, environmental and personal health interface. | •      Support use of informatics to promote disease prevention at the clinical health, environmental and personal health interface. | •      Support use of informatics to promote disease prevention at the clinical health, environmental and personal health interface. |  |
|  | **C.3** | •      Ensure that knowledge, information, and data needs of project or program users and stakeholders are met.​ | •      Ensure that knowledge, information, and data needs of project or program users and stakeholders are met.​ | •      Leads interdisciplinary teams to ensure that knowledge, information, and data needs of project or program users and stakeholders are met.​ | •      Understands and communicates knowledge, information, and data needs of project or program users and stakeholders are met.​ |  |
|  | **C.4** | •      Design, develop and implement person-centered population health information systems. | •      Design, develop and implement person-centered population health information systems. | •      Understands and leads the design, development and implementation of person-centered population health information systems. | •      Understands the importance of person-centered population health information systems and is aware of the processes involved in creating these systems. |  |
|  | **C.5** | •      Support information system development, procurement, and implementation that meet public health program needs.​ | •      Leads information system development, procurement, and implementation that meet public health program needs.​ | •      Leads interdisciplinary teams to support information system development, procurement, and implementation that meet public health program needs.​ | •      Support information system development, procurement, and implementation that meet public health program needs.​ |  |
|  | **C.6** | •      Establish frameworks for evaluating the implementation process of information systems and applications and make recommendations to improve user satisfaction and outcomes. | •      Creates and applies frameworks for evaluating the implementation process of information systems and applications and make recommendations to improve user satisfaction and outcomes. | •      Understands frameworks for evaluating the implementation process of information systems and applications and make recommendations to improve user satisfaction and outcomes. | •      Is aware of frameworks for evaluating the implementation process of information systems and applications and make recommendations to improve user satisfaction and outcomes. |  |
|  | **C.7** | •      Evaluate communication - Assess reach and dose of communication using tools (e.g., website and social media analytics, data mining software, focus groups, in-depth interviews). | •      Evaluate communication - Assess reach and dose of communication using tools (e.g., website and social media analytics, data mining software, focus groups, in-depth interviews). | •      Understands methods for evaluating communication - e.g., assessing the reach and dose of communication using tools (e.g., website and social media analytics, data mining software, focus groups, in-depth interviews). | •      Understands methods for evaluating communication - e.g., assessing the reach and dose of communication using tools (e.g., website and social media analytics, data mining software, focus groups, in-depth interviews). |  |
|  | **C.8** | •      Develop team approaches that bring together diverse disciplines and organizations to develop new and creative ways of designing and implementing studies and addressing public health concerns. | •      Develop team approaches that bring together diverse disciplines and organizations to develop new and creative ways of designing and implementing studies and addressing public health concerns. | •      Develop team approaches that bring together diverse disciplines and organizations to develop new and creative ways of designing and implementing studies and addressing public health concerns. | •      Understands team approaches to build diverse teams to design and implement studies and address public health concerns. |  |
| **Partnerships, collaboration, and advocacy** | **D.1** | •      Assess stakeholder data, information, and knowledge needs. | •      Assess stakeholder data, information, and knowledge needs. | •      Assess stakeholder data, information, and knowledge needs. | •      Assess stakeholder data, information, and knowledge needs. |  |
|  | **D.2** | •      Use new media to conduct advocacy e.g., social media. | •      Use new media to conduct advocacy e.g., social media. | •      Use new media to conduct advocacy e.g., social media. | •      Use new media to conduct advocacy e.g., social media. |  |
|  | **D.3** | •      Manage IT operations related to project or program and those managed by external organizations. | •      Manage IT operations related to project or program and those managed by external organizations. | •      Understands IT operations related to project or program and those managed by external organizations. | •      Is aware of IT operations related to project or program and those managed by external organizations. |  |
|  | **D.4** | •      Develop team approaches that bring together diverse disciplines and organizations to develop new and creative ways of designing and implementing studies and addressing public health concerns. | •      Develop team approaches that bring together diverse disciplines and organizations to develop new and creative ways of designing and implementing studies and addressing public health concerns. | •      Develop team approaches that bring together diverse disciplines and organizations to develop new and creative ways of designing and implementing studies and addressing public health concerns. | •      Develop team approaches that bring together diverse disciplines and organizations to develop new and creative ways of designing and implementing studies and addressing public health concerns. |  |
| **Diversity and inclusion** | **E.1** | •      Effectively communicate with teams through emails, word processing, spreadsheet, and presentation software. | •      Effectively communicate with teams through emails, word processing, spreadsheet, and presentation software. | •      Effectively communicate with teams through emails, word processing, spreadsheet, and presentation software. | •      Effectively communicate with teams through emails, word processing, spreadsheet, and presentation software. |  |
| **Communication** | **F.1** | •      Use evidence-based communication program models to disseminate research and evaluation outcomes. | •      Use evidence-based communication program models to disseminate research and evaluation outcomes. | •      Understands evidence-based communication program models and leads teams to use these models to disseminate research and evaluation outcomes. | •      Contributes to the use of evidence-based communication program models to disseminate research and evaluation outcomes. |  |
|  | **F.2** | •      Use mass media, electronic technology, and communication methods (e.g., social media, social) for public health communication, health education and promotion. | •      Use mass media, electronic technology, and communication methods (e.g., social media, social) for public health communication, health education and promotion. | •      Use mass media, electronic technology, and communication methods (e.g., social media, social) for public health communication, health education and promotion. | •      Use mass media, electronic technology, and communication methods (e.g., social media, social) for public health communication, health education and promotion. |  |
|  | **F.3** | •      Use information technology to assure openness of public health agency processes and responsiveness to the public. | •      Use information technology to assure openness of public health agency processes and responsiveness to the public. | •      Understand the use of information technology in public communication and lead teams to use technology to assure openness of public health agency processes and responsiveness to the public. | •      Understands the use of information technology to assure openness of public health agency processes and responsiveness to the public. |  |
|  | **F.4** | •      Define target audience, develop correct messaging to reach people where they are, considering the people, places, and media they interact with daily and the information sources and formats they trust. | •      Define target audience, develop correct messaging to reach people where they are, considering the people, places, and media they interact with daily and the information sources and formats they trust. | •      Understand methods for delivering targeted public health messaging to reach people where they are, considering the people, places, and media they interact with daily and the information sources and formats they trust. | •      Is aware of methods for delivering targeted public health messaging to reach people where they are, considering the people, places, and media they interact with daily and the information sources and formats they trust. |  |
|  | **F.5** | •      Tailor surveillance information content and periodicity of dissemination for specific audiences and their uses. | •      Tailor surveillance information content and periodicity of dissemination for specific audiences and their uses. | •      Understand the use of tailored surveillance information content and periodically disseminated health information for specific audiences. | •      Is aware of the use of tailored surveillance information content and periodically disseminated health information for specific audiences. |  |
|  | **F.6** | •      Evaluate communication - Assess reach and dose of communication using tools (e.g., website and social media analytics, data mining software, focus groups, in-depth interviews). | •      Evaluate communication - Assess reach and dose of communication using tools (e.g., website and social media analytics, data mining software, focus groups, in-depth interviews). | •      Understands methods for evaluating communication - e.g., assessing the reach and dose of communication using tools (e.g., website and social media analytics, data mining software, focus groups, in-depth interviews). | •      Understands methods for evaluating communication - e.g., assessing the reach and dose of communication using tools (e.g., website and social media analytics, data mining software, focus groups, in-depth interviews). |  |
|  | **F.7** | •      Support development of strategic direction for public health informatics within the enterprise. | •      Support development of strategic direction for public health informatics within the enterprise. | •      Lead the development of strategic direction for public health informatics within the enterprise. | •      Contribute (where possible) to the development of strategic direction for public health informatics within the enterprise. |  |
| **Leadership** | **G.1** | •      Show entrepreneurial orientation through proactiveness, innovativeness and risk-taking to advance public health, address newly emerging public health issues and better understand risks when aligning community health partners to address health inequities. | •      Show entrepreneurial orientation through proactiveness, innovativeness and risk-taking to advance public health, address newly emerging public health issues and better understand risks when aligning community health partners to address health inequities. | •      Show entrepreneurial orientation through proactiveness, innovativeness and risk-taking to advance public health, address newly emerging public health issues and better understand risks when aligning community health partners to address health inequities. | •      Show entrepreneurial orientation through proactiveness, innovativeness and risk-taking to advance public health, address newly emerging public health issues and better understand risks when aligning community health partners to address health inequities. |  |
|  | **G.2** | •      Address IT development using principles of systems thinking and engineering. | •      Address IT development using principles of systems thinking and engineering. | •      Understand IT development and lead teams to support the process using principles of systems thinking and engineering. | •      Is aware of IT development using principles of systems thinking. |  |
|  | **G.3** | •      Communicate PHI needs and proposed solutions to leaders in public health and elected officials effectively. | •      Communicate PHI needs and proposed solutions to leaders in public health and elected officials effectively. | •      Communicate PHI needs and proposed solutions to leaders in public health and elected officials effectively. | •      Understand PHI needs and proposed solutions. |  |
|  | **G.4** | •      Contribute to the consideration of the best IT systems to use by the enterprise. | •      Contribute to the consideration of the best IT systems to use by the enterprise. | •      Contribute to the consideration of the best IT systems to use by the enterprise. | •      Contribute to the consideration of the best IT systems to use by the enterprise (where possible). |  |
|  | **G.5** | •      Manage and direct health informatics planning for projects related to public health and information technology. | •      Manage and direct health informatics planning for projects related to public health and information technology. | •      Understand and direct health informatics planning for projects related to public health and information technology. | •      Is aware of health informatics planning processes for projects related to public health and information technology. |  |
|  | **G.6** | •      Apply procedures (policies) and technical means (security) to ensure integrity and protection of confidential information in electronic files and computer systems while maximizing the benefits to public health. | •      Apply procedures (policies) and technical means (security) to ensure integrity and protection of confidential information in electronic files and computer systems while maximizing the benefits to public health. | •      Apply procedures (policies) and technical means (security) to ensure integrity and protection of confidential information in electronic files and computer systems while maximizing the benefits to public health. | •      Apply procedures (policies) and technical means (security) to ensure integrity and protection of confidential information in electronic files and computer systems while maximizing the benefits to public health. |  |
| **Data and data systems management and governance** | **H.1** | •      Develop tools that protect the privacy of individuals and communities involved in health programs, policies, and research. | •      Develop and apply tools that protect the privacy of individuals and communities involved in health programs, policies, and research. | • Understand processes for developing and applies tools that protect the privacy of individuals and communities involved in health programs, policies, and research. | •      Is aware and applies tools that protect the privacy of individuals and communities involved in health programs, policies, and research. |  |
|  | **H.2** | •      Contribute to development of public health information systems that are interoperable with other relevant information systems.​ | •      Develop public health information systems that are interoperable with other relevant information systems.​ | •      Understands and leads development of public health information systems that are interoperable with other relevant information systems.​ | •      Contribute to development of public health information systems that are interoperable with other relevant information systems.​ |  |
|  | **H.3** | •      Manage data and information in compliance with policies, protocols, and informatics standards. | •      Manage data and information in compliance with policies, protocols, and informatics standards. | •      Understand data and information management policies, protocols, and informatics standards. | •      Understand data and information management policies, protocols, and informatics standards. |  |
|  | **H.4** | •      Aid public health organizations in thinking through and designing testing strategies for new systems. | •      Design, test and implement strategies for new digital information systems. | •      Lead teams to design, test and implement strategies for new digital information systems. | •      Contribute to the design, testing strategies for new systems. |  |
|  | **H.5** | •      Describe systems analysis methodology, requirements, use cases, data flow diagrams, business process modeling, data modeling, relational data theory, normalization, entity relationship diagrams, SQL, and data warehouses. | •      Describe and apply systems analysis methodology, requirements, use cases, data flow diagrams, business process modeling, data modeling, relational data theory, normalization, entity relationship diagrams, SQL, and data warehouses. | •      Understand systems analysis methodology, requirements, use cases, data flow diagrams, business process modeling, data modeling, relational data theory, normalization, entity relationship diagrams, SQL, and data warehouses. | •      Is aware of systems analysis methodology, requirements, use cases, data flow diagrams, business process modeling, data modeling, relational data theory, normalization, entity relationship diagrams, SQL, and data warehouses. |  |
|  | **H.6** | •      Document business rules using algorithms and pseudocode. | •      Document business rules using algorithms and pseudocode. | •      Understands processes for documenting business rules using algorithms and pseudocode. | •     Is aware of business rules regarding data and information systems. |  |
|  | **H.7** | •      Assess and manage risks associated with using and sharing data and information, data security and intellectual property. | •      Assess and manage risks associated with using and sharing data and information, data security and intellectual property. | •      Assess and manage risks associated with using and sharing data and information, data security and intellectual property. | •      Assess and manage risks associated with using and sharing data and information, data security and intellectual property. |  |
|  | **H.8** | •      Describe conceptualizations of data, medical vocabulary nomenclature, terminologies, coding and classification, standards ethics, privacy, security, health systems, computer technologies, health information exchange, data quality, and information architectures. | •      Apply conceptualizations of data, medical vocabulary nomenclature, terminologies, coding and classification, standards ethics, privacy, security, health systems, computer technologies, health information exchange, data quality, and information architectures. | •      Understands conceptualizations of data, medical vocabulary nomenclature, terminologies, coding and classification, standards ethics, privacy, security, health systems, computer technologies, health information exchange, data quality, and information architectures. | •      Is aware of conceptualizations of data, medical vocabulary nomenclature, terminologies, coding and classification, standards ethics, privacy, security, health systems, computer technologies, health information exchange, data quality, and information architectures. |  |
|  | **H.9** | •      Construct transactions for health information exchange. | •      Construct transactions for health information exchange. | •      Understands processes for constructing transactions for health information exchange. | •      Is aware of health information exchange systems and protocols. |  |
|  |  |  |  |  |  |  |

^$^Adaptations to tiered competency statements made based on participants’ comments; ^*^ Competency statement broken into two simpler statements; IT: Information technology; PHI: Public Health Informatics; SQL: Structured Query Language; MS-SQL: Microsoft Structured Query Language

**Appendix 3 - Consolidated criteria for reporting qualitative studies (COREQ): 32-item checklist**

Developed from:

Tong A, Sainsbury P, Craig J. Consolidated criteria for reporting qualitative research (COREQ): a 32-item checklist for interviews and focus groups. *International Journal for Quality in Health Care*. 2007. Volume 19, Number 6: pp. 349 – 357

| **No. Item** | **Guide questions/description** | **Reported on Page #** |
| --- | --- | --- |
| **Domain 1: Research team and reﬂexivity** |  |  |
| *Personal Characteristics* |  |  |
| 1. Interviewer/facilitator | Which author/s conducted the interview or focus group? | 9-10 |
| 2. Credentials | What were the researcher’s credentials? E.g., PhD, MD | 9-10 |
| 3. Occupation | What was their occupation at the time of the study? | 9-10 |
| 4. Gender | Was the researcher male or female? | 9-10 |
| 5. Experience and training | What experience or training did the researcher have? | 9-10 |
| *Relationship with participants* |  |  |
| 6. Relationship established | Was a relationship established prior to study commencement? | 9-10 and Appendix |
| 7. Participant knowledge of the interviewer | What did the participants know about the researcher? e.g., personal goals, reasons for doing the research | 9-10 and Appendix |
| 8. Interviewer characteristics | What characteristics were reported about the inter viewer/facilitator? e.g., Bias, assumptions, reasons and interests in the research topic | 7-8 |

| **Domain 2: study design** |  |  |
| --- | --- | --- |
| *Theoretical framework* |  |  |
| 9. Methodological orientation and Theory | What methodological orientation was stated to underpin the study? e.g. grounded theory, discourse analysis, ethnography, phenomenology, content analysis | 7-8 |
| *Participant selection* |  |  |
| 10. Sampling | How were participants selected? e.g. purposive, convenience, consecutive, snowball | 8 |
| 11. Method of approach | How were participants approached? e.g. face-to-face, telephone, mail, email | 8 |
| 12. Sample size | How many participants were in the study? | 9 and Table 1 |
| 13. Non-participation | How many people refused to participate or dropped out? Reasons? | NA |
| *Setting* |  |  |
| 14. Setting of data collection | Where was the data collected? e.g. home, clinic, workplace | 9-10 |
| 15. Presence of non-participants | Was anyone else present besides the participants and researchers? | 9-10 |
| 16. Description of sample | What are the important characteristics of the sample? e.g. demographic data, date | 12 and Table 2 |
| *Data collection* |  |  |
| 17. Interview guide | Were questions, prompts, guides provided by the authors? Was it pilot tested? | Appendix |
| 18. Repeat interviews | Were repeat inter views carried out? If yes, how many? | NA |
| 19. Audio/visual recording | Did the research use audio or visual recording to collect the data? | 9-10 |
| 20. Field notes | Were ﬁeld notes made during and/or after the interview or focus group? | 9-10 |
| 21. Duration | What was the duration of the inter views or focus group? | 9-10 |
| 22. Data saturation | Was data saturation discussed? | NA (data saturation not required in interpretive description) |
| 23. Transcripts returned | Were transcripts returned to participants for comment and/or correction? | NA |
| **Domain 3: analysis and ﬁndings** |  |  |
| *Data analysis* |  |  |
| 24. Number of data coders | How many data coders coded the data? | 11 |
| 25. Description of the coding tree | Did authors provide a description of the coding tree? | 15 |
| 26. Derivation of themes | Were themes identiﬁed in advance or derived from the data? | 11-12 |
| 27. Software | What software, if applicable, was used to manage the data? | 11 |
| 28. Participant checking | Did participants provide feedback on the ﬁndings? | NA (advisory group of researchers used instead) |
| *Reporting* |  |  |
| 29. Quotations presented | Were participant quotations presented to illustrate the themes/ﬁndings? Was each quotation identiﬁed? e.g., participant number | 16-22 |
| 30. Data and ﬁndings consistent | Was there consistency between the data presented and the ﬁndings? | 16-22 |
| 31. Clarity of major themes | Were major themes clearly presented in the ﬁndings? | 16-22 |
| 32. Clarity of minor themes | Is there a description of diverse cases or discussion of minor themes? | 16-22 |
